# Complete Heart Block as a Clinical Feature in Critically Ill Coronavirus Disease 2019 (COVID-19) Patients: A Case Series of Three Cases

**DOI:** 10.1155/2021/9955466

**Published:** 2021-08-12

**Authors:** Farook Ahmad, Priti Gandre, Julien Nguekam, Alanna Wall, ShiYu Ong, Abdul N. Karuppamakkantakath, Konstantinos Tasopoulos, Muhammad Athar Sadiq, Sazzli Kasim, Jeronimo M. Cuesta

**Affiliations:** ^1^Critical Care Unit, North Middlesex University, Hospital NHS Trust, Sterling Way, London N18 1QX, UK; ^2^Cardiology Unit, Medical Department of Universiti Teknologi MARA (UiTM) Sungai Buloh, Malaysia; ^3^Cardiology Unit, Department of Medicine, Sultan Qaboos University, Muscat 123, Oman

## Abstract

*Background*. Novel coronavirus-19 disease (COVID-19) is associated with significant cardiovascular morbidity and mortality. However, there have been very few reports on complete heart block (CHB) associated with COVID-19. This case series describes clinical characteristics, potential mechanisms, and short-term outcomes of critically ill COVID-19 patients complicated by CHB. *Case Summary*. We present three cases of new-onset CHB in critically ill COVID-19 patients. Patient 1 is a 41-year-old male with well-documented history of Familial Mediterranean Fever (FMF) who required mechanical ventilator support for acute hypoxic respiratory failure from severe COVID-19 pneumonia. He developed new-onset CHB without a hemodynamic derangement but subsequently had acute coronary syndrome complicated by cardiogenic shock. Patient 2 is a 77-year-old male with no past medical history who required intubation for severe COVID-19 pneumonia acute hypoxic respiratory failure. He developed CHB with sinus pause requiring temporary pacing but subsequently developed multiorgan failure. Patient 3 is 36-year-old lady 38 + 2 weeks pregnant, gravida 2 para 1 with no other medical history, who had an emergency Lower Section Caesarean Section (LSCS) as she required intubation for acute hypoxic respiratory failure. She exhibited new-onset CHB without hemodynamic compromise. The CHB resolved spontaneously after 24 hours. *Discussion*. COVID-19-associated CHB is a very rare clinical manifestation. The potential mechanisms for CHB in patients with COVID-19 include myocardial inflammation or direct viral infiltration as well as other causes such as metabolic derangements or use of sedatives. Patients diagnosed with COVID-19 should be monitored closely for the development of bradyarrhythmia and hemodynamic instability.

## 1. Introduction

In January 2020, the World Health Organization declared the outbreak of novel coronavirus, SARS-CoV-2 (COVID-19) a Public Health Emergency of International Concern [1, 2]. The primary clinical manifestation of COVID-19 is the respiratory system, but involvement of other systems has been reported, especially the cardiovascular system [1–3]. Cardiovascular complications of COVID-19 include myocardial infarction, myocarditis, heart failure, cardiogenic shock, and cardiac arrhythmias [3]. However, CHB is a very rare cardiovascular complication of COVID-19 disease. We present a case series of 3 patients with no prior cardiovascular history and otherwise normal cardiac structure and function who were admitted to the Intensive Care Unit with COVID-19 and developed CHB during their clinical course. We believe that this is a rare yet fatal cardiovascular complication of COVID-19 disease. Thus, it is important for clinicians to be aware of this severe manifestation of the disease.

## 2. Case 1

Patient 1 was a 41-year-old businessman, with history of Familial Mediterranean Fever (FMF) on long-term colchicine, who presented to our institution with 1 week of fever associated with pleuritic chest pain and breathlessness. On arrival, he was noted to be hypotensive with elevated jugular venous pressure as well as severe respiratory distress requiring mechanical ventilator support. A nasopharyngeal swab test for SARS-CoV-2 by polymerase chain reaction was positive, and he was treated with Tocilizumab and Dexamethasone as per institutional practice. He was further managed in the Intensive Care Unit (ICU) for COVID-19 pneumonitis. Initial laboratory investigations showed lymphopenia (688 cells/mm^3^) and raised inflammatory markers (CRP 110 mg/L, fibrinogen 888 mg/dL, and D-dimer 11000 ng/mL Fibrinogen Equivalent Units (FEU)). His renal markers, electrolytes, and troponin T on admission were all within normal limits (Table 1, Supplementary Data). Electrocardiogram (ECG) on presentation showed normal sinus rhythm at a rate of 82 beats per minute (bpm), normal intervals, normal axis, and no ST-T wave changes. There was no previous ECG for comparison.

**Figure 1:**
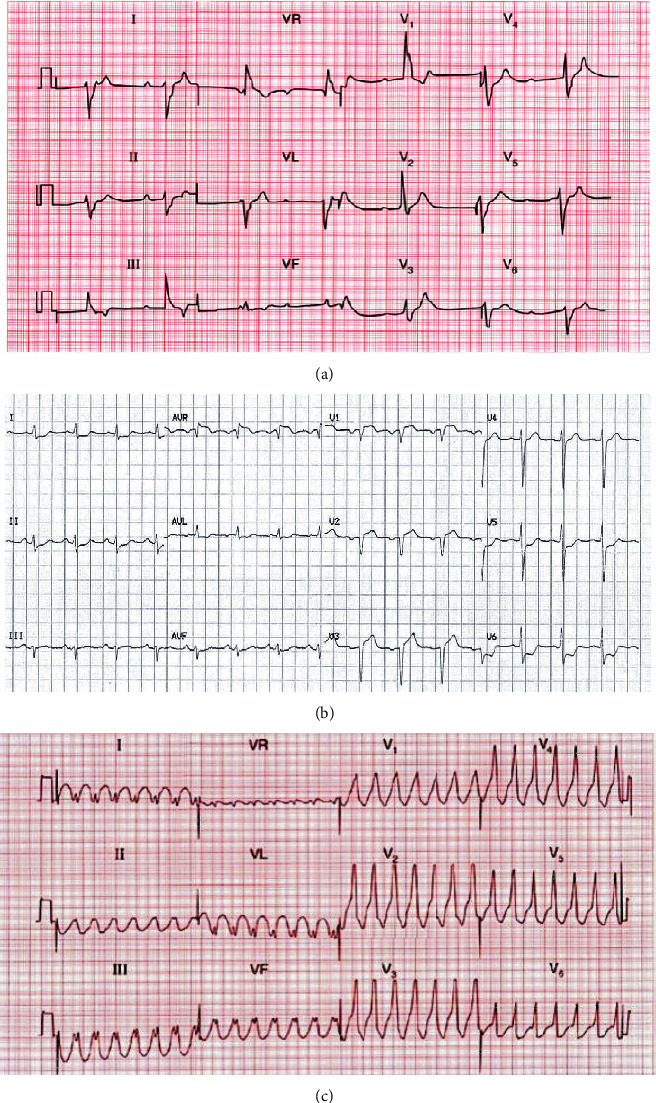
(a) ECG of patient 1 on day 5 ICU admission showing CHB with ventricular escape rhythm rate approximately 50 bpm. (b) ECG of patient 1 on day 8 admission showing ST elevation in lead AVR and ST depression at leads 1, AVL, II, AVF, V5, and V. (c) ECG of patient 1 on day 8 admission showing ventricular tachycardia and subsequently pulseless electrical activity (not recorded).

He was proned immediately after endotracheal intubation. On day 5 of ICU admission, the patient was noted to be in complete heart block on cardiac telemetry monitoring.  Figure 1(a) shows that a 12-lead electrocardiogram performed confirmed the diagnosis of CHB with a ventricular rate of approximately 50 bpm. Immediate laboratory investigations including thyroid function, cardiac biomarkers, and electrolytes were unremarkable. Echocardiogram revealed a normal left ventricular ejection fraction with no regional wall motion abnormalities or valvular pathology. The ongoing medication list was checked and found to be unremarkable for precipitating atrioventricular (AV) block. The complete heart block continued with a spontaneous and stable ventricular rate of 40-45 beats per minute with stable hemodynamics for a further three days. It was managed conservatively under vigilant monitoring with invasive cardiac output monitoring. On day 8 of ICU admission, he acutely deteriorated and had cardiogenic shock requiring noradrenaline and adrenaline. ECG showed ST elevation in AVR with global ST depression (Figure 1(b)). Cardiac troponins were significantly raised, and a repeat 2-D echocardiogram showed global hypokinesia with severely reduced ejection fraction of 20%. Shortly after, the patient deteriorated further and developed ventricular tachycardia (Figure 1(c)) and passed away later that day following a failed cardiopulmonary resuscitation (CPR). A timeline event for this patient is provided in Table 2, Supplementary data.

## 3. Case 2

Patient 2 is a 77-year-old Italian man with no significant past medical history who was brought in by ambulance to the emergency department with acute onset of shortness of breath for 3-day duration. He was noted to be severely hypoxic with SpO_2_ < 50% upon arrival of paramedics at his residential home. He was intubated and proned immediately upon arrival from the emergency department. A nasopharyngeal swab was positive for SARS-CoV-2 by polymerase chain reaction and was treated with Tocilizumab and Dexamethasone as per institutional practice. Initial laboratory workup showed lymphopenia (628 cells/mm^3^) and elevated inflammatory markers (CRP 128 mg/L, fibrinogen 818 mg/dL, and D-dimer 17000 ng/mL FEU). His other laboratory workup was within normal limits, including a normal troponin T (Table 1, Supplementary Data). His initial ECG showed a normal sinus rhythm with heart rate of 94 bpm, but no other abnormalities were found. There was no previous ECG for comparison. Chest radiography revealed bilateral ground-glass opacities and computed tomography pulmonary angiogram (CTPA) showed extensive patchy peripheral ground-glass opacification throughout the thorax but no evidence of pulmonary embolism. Echocardiogram revealed a normal left ventricular ejection fraction with no regional wall motion abnormalities or valvular pathology.

**Figure 2:**
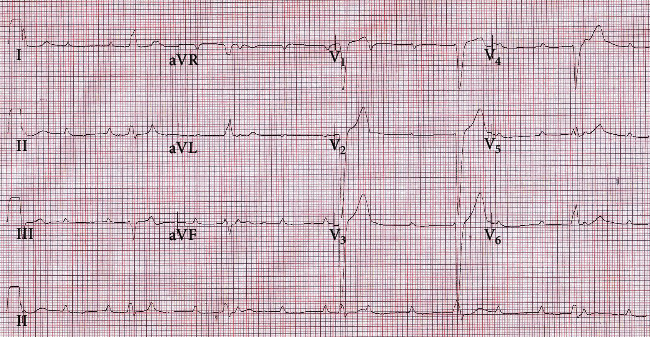
ECG for patient 2 on day 7 of admission showing CHB with ventricular rate approximately 35 bpm.

The clinical course in ICU was complicated by shock on day 7 of admission which progressed into multiorgan failure and support requiring continuous renal replacement therapy and hepatic failure as well as elevated troponin level (peak troponin T level was 5003 ng/L). He was also noted to have bradycardia with cardiac telemetry monitoring which revealed a CHB with occasional sinus pause, and the 12-lead ECG showed a CHB (Figure 2). These episodes of AV block alternating with CHB continued with increasing inotropic support. Laboratory investigations for electrolytes and thyroid function were all within normal limits. A balloon flotation temporary pacing wire was inserted successfully using bedside echocardiogram guidance, and he was scheduled for permanent pacemaker implantation. Unfortunately, he developed refractory hypoxic respiratory failure and was deemed not a suitable candidate for Extracorporeal Membrane Oxygenation (ECMO). Successive family conferences resulted in family shifting the focus of care to comfort measures. He passed away on day 10 of admission. A timeline event for this patient is provided in Table 2, Supplementary data.

## 4. Case 3

**Figure 3:**
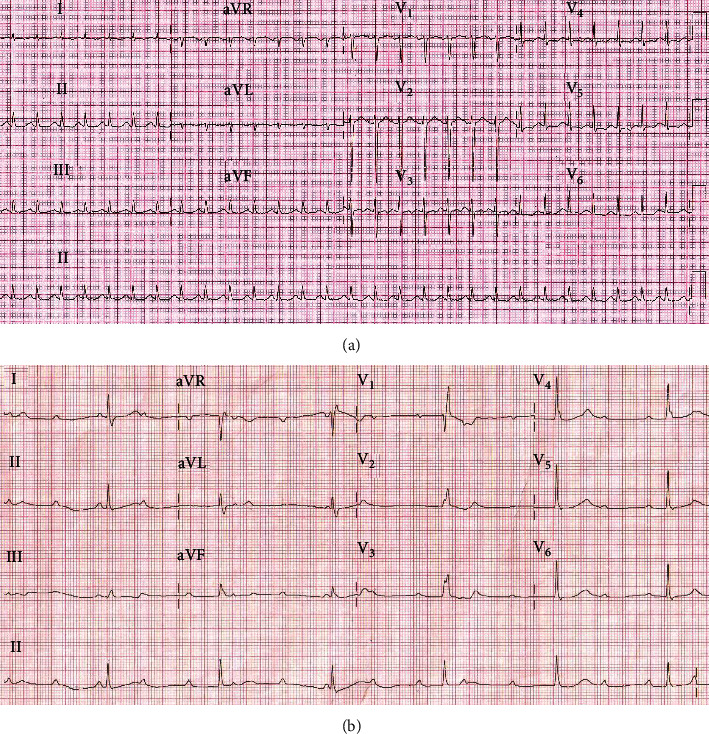
(a) ECG on admission for patient 3 showing sinus tachycardia. (b) ECG for patient 3 on day 5 admission showing complete heart block CHB with narrow QRS, suggesting distal AV node focus as origin of escape rhythm.

Patient 3 is a 36-year-old pregnant female, gravida 2 para 1 (G2P1) with previous Lower Segment Caesarean Section (LSCS) and currently at 38 + 2 weeks pregnant presented with acute onset of shortness of breath for 4-day duration associated with fever, myalgias, and diarrhea for 4-day duration. On arrival to the emergency department, the patient was noted to be febrile with a temperature of 38.8°C, tachycardic with a heart rate of 105 bpm, respiratory rate of 32 breaths per minute, and severely hypoxic with an oxygen saturation of 82% on room air. Her nasopharyngeal swab tested positive for SARS-CoV-2 by polymerase chain reaction and was treated with Tocilizumab and Dexamethasone as per institutional practice. Chest radiography revealed bilateral infiltrates. Initial lab workup revealed mildly elevated white blood cell count with lymphopenia (700 cells/mm^3^), elevated procalcitonin (6.4 ng/mL), LDH (610 U/L), D-dimer 14000 ng/mL FEU, but normal troponin T < 0.01 ng/mL (Table 1, Supplementary data). ECG on admission (Figure 3(a)) showed sinus tachycardia at 167 bpm, normal intervals, normal axis, and absence of ischemic findings. She was commenced on noninvasive ventilation and had CTPA that showed severe extensive bilateral patchy ground-glass opacification throughout but no evidence of pulmonary embolism. She was intubated for worsening hypoxic respiratory failure and underwent a LSCS with no further complication and was subsequently admitted to ICU.

Her course of admission was complicated by progressive hypoxemia requiring prone ventilation. On day 3, the patient was noted to be in shock requiring inotropic support. Her ECG showed sinus tachycardia with diffuse T wave inversion. Echocardiogram showed global hypokinesia with depressed ejection fraction of 30% but normal septal thickness of 1.2 cm. Her serum CK was elevated at 600 U/L. A diagnosis of myocarditis was made [4], and she was continued on supportive care.

On day 5 of admission to ICU, intermittent complete heart block was seen on telemetry for less than 24 hours and 12-lead ECG showed a CHB with narrow QRS, suggesting distal AV node focus as origin of escape rhythm (Figure 3(b)). However, she was managed conservatively with vigilant monitoring as she was receiving prone ventilation for refractory hypoxia at the time and she was stable on single, nonescalating inotropic support. Her laboratory investigations for electrolytes and thyroid function were all within normal range. The CHB resolved gradually with improvement in hypoxemia. She was deemed not a suitable candidate for ECMO as the pAO_2_/FiO_2_ ratio improved with proning maneuvers. A repeat echocardiography shows improved ejection fraction to 50%. The patient was subsequently discharged to another critical care facility for continuation in management and was stable at the time of writing this report. She was also planned for repeat echocardiography as outpatient cardiology clinic follow-up. A timeline event for this patient is provided in Table 2, Supplementary data.

## 5. Discussion

The SARS-CoV-2 virus has caused a worldwide pandemic infecting millions of people, targeting primarily the respiratory system [1–3]. Cardiovascular complications have been reported with varying incidences as one of the manifestations that confer a worse prognosis [4]. Possible etiologies for cardiovascular complications include hypoxemia, electrolyte and metabolic abnormalities, microthrombi, and/or cardiomyopathy from direct invasion to the cardiomyocytes. Cardiac arrhythmias affecting the atrioventricular (AV) node have been reported in association with cardiac injury in critically ill COVID-19 patients [4, 5].

In our case series, initial and subsequent troponin levels were normal for all the patients. In addition, echocardiography results were not suggestive of underlying cardiomyopathy. This indicates a new onset AV node disease, from either direct infiltration of the AV node, i.e., focused involvement of the AV node, or the AV block being secondary to the critical illness itself. Possible etiologies for new-onset AV node disease include coronary artery disease (CAD); functional, structural, or valvular heart diseases; thyroid disease; medications; and infections [6–8]. Thus, the escape rhythm tended to be narrow, similar to the sinus in morphology, and of adequate rate to support normal hemodynamics without requiring temporary pacing as seen in patient no. 3. The hemodynamic instability requiring temporary pacing in patient 2 is probably contributed by multiple underlying causes rather than the complete heart block alone. Patient 1, meanwhile, has well-documented history of FMF. It has been previously reported that FMF patients are prone to develop CAD, mostly due to increased inflammatory activity and endothelial dysfunction [9]. Several studies and case reports have described the pericardial involvement in FMF patients including acute and recurrent pericarditis, constrictive pericarditis, pericardial effusion, and cardiac tamponade [10]. However, there is no report to suggest a direct correlation between COVID-19 and FMF with cardiac arrhythmia especially CHB and/or acute coronary syndrome.

The implication of the development of AV block in critically ill COVID-19 patients remains undetermined. In this case series, we have had mixed outcomes from the new onset of AV node disease. The resolution of AV block in patient 3 without further intervention suggests that a temporary or even permanent pacemaker is not required, while patient 2 required immediate temporary pacing wire insertion. Often the prone positioning and clinical status of these exceptional diagnoses make insertion of pacemaker very difficult, which otherwise would be the primary option to treat complete heart block.

While in the midst of the pandemic, we have also noted that sinus bradycardia is a very common finding in critically ill COVID-19 patients. However, these cases were not reported due to the fact that sinus bradycardia commonly occurs in patients with mechanical ventilator support and concurrently on high doses of sedatives and NMBA agent.

Previous publications on cardiovascular manifestation of critically ill COVID-19 mostly focus on acute coronary syndrome and/or myocarditis. To our knowledge, atrioventricular block is a very rare clinical manifestation of COVID-19 patients. Thus, it is important for clinicians to be aware of such a possible severe manifestation with undesirable outcome. We hope that this series will lead to future investigations of the pathophysiology of COVID-19 on the cardiovascular conduction system.

## 6. Conclusion

CHB can be a clinical manifestation of critically ill COVID-19 patients. Vigilant monitoring and due clinical suspicion are necessary to detect this in a timely manner. The prognosis of the AV node involvement remains unknown and will need future investigations as well as long-term follow-up.

## Data Availability

Raw data or images were generated at North Middlesex University Hospital. Derived data supporting the findings of this study are available from the corresponding author (FA) on request.

## Supplementary Materials

Supplementary Materials Table 1: normal reference range for basic laboratory investigations. Table 2: event timeline for patients 1, 2, and 3.
