# Supplementary material for: Complete Heart Block as a Clinical Feature in Critically Ill Coronavirus Disease 2019 (COVID-19) Patients: A Case Series of Three Cases
Source: Case Rep Crit Care. 2021 Aug 12;2021:9955466. doi: 10.1155/2021/9955466 (PMC8376443; doi:10.1155/2021/9955466)
Supplement: Supplementary Materials — Table 1: normal reference range for basic laboratory investigations. Table 2: event timeline for patients 1, 2, and 3. [file 9955466.f1.docx]

**Supplemental Document**

Table 1: Normal Reference range for basic laboratory investigations

| Components | Normal reference range |
| --- | --- |
| Hemoglobin (g/L) | 130-150 |
| White Cell Count (10^9^/L) | 4-11 |
| Platelet (10^9^/L) | 150-450 |
| Lymphocytes (10^9^/L) | 1.5-4.5 |
| Creatinine (umol/L) | 50-110 |
| Urea (mmol/L) | 1.7-8.3 |
| Potassium (mmol/L) | 3.5-5 |
| Sodium (mmol/L) | 135-145 |
| Cardiac Troponin T (ng/L) | 0-14 |
| D-Dimer (ug/L FEU) | 0-550 |

**Table 2: Event Timeline for patient 1, 2 and 3**

| Days of Hospitalization | Event |
| --- | --- |
| Patient 1 | |
| Day 0 | Presented to Emergency department with 1 week of fever, pleuritic chest pain and breathlessness. Covid -19 is diagnosed. Intubated in Emergency Department |
| Day 1 | Deprone  Transthoracic echocardiogram does not demonstrate any structural, valvular or functional abnormalities. |
| Day 3 | Developed sinus bradycardia with heart rate mostly between 55-60. |
| Day 5 | Developed CHB withour hemodynamics instability. |
| Day 8 | Developed ACS with cardiogenic shock and then pulse ventricular tachycardia followed by Pulseless Electrical Activity (PEA) and asystole. Resuscitated as per ACLS protocol but patient remained asystole. Pronounced dead |
| Patient 2 | |
| Day 0 | Presented to Emergency Department with acute onset of shortness of breath of 3 days duration duration with severe hypoxic respiratory failure. Intubated in Emergency Department. Covid -19 is diagnosed. |
| Day 1 | Deprone |
| Day 5 | Worsening PaO2/FiO2 ratio. Had Computed Tomography pulmonary angiogram (CTPA) that showed extensive patchy peripheral ground-glass opacification throughout the thorax but no pulmonary embolism. |
| Day 6 | Developed oligouric acute kidney injury (AKI) and MAP < 60. Started on Noradrenaline. |
| Day 7 | Deteriorate to anuria AKI and started on Continuous Renal Replacement Therapy (CRRT). Developed CHB requiring 2 inotropes.  Temporary pacing wire inserted. Bedside echocardiography showed no pericardial effusion |
| Day 8 | Developed refractory hypoxemia with severe Pao2/FiO2 ratio. Chest Radiograph is not suggestive of pneumothorax. No pericardial effusion on Repeat bedside echocardiography |
| Day 10 | Family opted for Comfort measures. Patient passed away |
| Patient 3 | |
| Day 0 | Presented to Emergency Department acute onset of shortness of breath for 4 days duration associated with fever, myalgias and diarrhea. Covid -19 is diagnosed. Started on Non Invasive ventilation. |
| Day 2 | Intubated for worsening hypoxic respiratory failure. Underwent Lower Section Caesarean Section (LSCS). Proned immediately after. |
| Day 3 | Deprone.  Map< 60. Started on noradrenaline. ECG, echocardiogram findings and laboratory investigations is suggestive of  Myocarditis. |
| Day 5 | Developed CHB while in prone position. |
| Day 6 | CHB resolved. PaO2/FiO2 ratio improved. |
| Day 8 | Transferred to other Critical Care Centre for continuation of care |
